# The Prevalence of Low Birth Weight Among Newborn Babies and Its Associated Maternal Risk Factors: A Hospital-Based Cross-Sectional Study

**DOI:** 10.7759/cureus.38587

**Published:** 2023-05-05

**Authors:** Amar Devaguru, Sandeep Gada, Dnyaneshwar Potpalle, Mummareddi Dinesh Eshwar, Dipti Purwar

**Affiliations:** 1 Paediatrics, People’s Hospital, Hyderabad, IND; 2 Paediatrics, Mahavir Institute of Medical Sciences, Vikarabad, IND; 3 Critical Care, Bombay Hospital, Mumbai, IND

**Keywords:** pregnancy, maternal risk factors, prevalence, neonates, low birth weight

## Abstract

Background

Low birth weight (LBW) is at the forefront of 100 core health issues that are used as indicators to assess the global nutrition monitoring framework as reported by the World Health Organization (WHO). Several factors could contribute to LBW, which essentially include intrauterine growth retardation and premature delivery/birth. Moreover, LBW predisposes neonates to several developmental disturbances including both physical and mental disorders. Given that LBW is more common in poor and developing countries, there is not much reliable data that could be used to formulate strategies for controlling this problem. This study, therefore, attempts to assess the prevalence of LBW among newborn babies and its associated maternal risk factors.

Methods

This hospital-based cross-sectional study was carried out between June 2016 and May 2017 (one year) and included 327 LBW babies. A predefined and prevalidated questionnaire was used to obtain data for the study. The data collected included age, religion, parity, birth spacing, pre-pregnancy weight, weight gain during pregnancy, height, mother’s education, occupation, family income, socioeconomic status, obstetric history, previous history of stillbirths and abortions, and history of any LBW baby.

Results

The prevalence of LBW was noted to be 36.33%. The occurrence of LBW babies was predominant among mothers who were aged <19 years (62.26%) and >35 years (57.14%). Grand multipara women showed the highest rates (53.70%) of LBW babies. Additionally, LBW was predominantly noticed among newborns (46.66%) with a birth spacing of <18 months, those born to mothers with pre-pregnancy weight of <40 Kg (94.04%), mothers with a height of <145 cm (83.46%), mothers who gained <7 kg during the pregnancy (82.20%), illiterate mothers (43.75%), and mothers who were agricultural workers (63.76%). Other maternal factors that could predispose to LBW included lower monthly income (66.25%), low socioeconomic status (52.90%), less number of antenatal visits (59.65%), low blood hemoglobin (100%), history of strenuous physical activities (48.66%), smoking and/or tobacco chewing habit (91.42%), alcoholism (66.66%), lack of iron and folic acid supplementation during pregnancy (64.58%), history of stillbirths (51.51%), and mothers suffering from chronic hypertension, preeclampsia, and eclampsia (47.61%), and tuberculosis (75%). Religion-wise, Muslim mothers revealed the highest prevalence (48.57%) of LBW, followed by Hindus (37.71%) and Christians (20%). The mother’s age, pre-pregnancy weight, weight gain during pregnancy, height of the mother, hemoglobin concentration, weight of the baby, and length of the newborn (p≤0.05) could influence the health of the newborn. However, maternal infections, previous bad obstetrics history, presence of systemic illnesses, and protein and calorie supplementation (p≥0.05) had no significant impact on birth weight.

Conclusions

The results showed that multiple factors are responsible for LBW. Maternal factors such as weight, height, age, parity, weight gained during pregnancy, and anemia during pregnancy could predispose to delivering LBW babies. Additionally, other risk factors for LBW identified in this study were the literacy level of mothers, occupation, family income, socioeconomic status, antenatal care, strenuous physical activity during pregnancy, smoking/tobacco chewing, alcohol/toddy consumption, and iron and folic acid supplementation during pregnancy.

## Introduction

Low birth weight (LBW) is one of the most serious challenges in maternal and child health, especially in developing countries such as India. The World Health Organization (WHO) defines LBW as a birth weight smaller than 2,500 grams irrespective of the period of gestation. Based on epidemiological observations, it was noticed that infants weighing less than 2,500 grams are approximately 20 times more likely to die than heavier babies [1].

The WHO has estimated that globally, about 20 million LBW babies are born each year, comprising 15.5% of all live births, and nearly 95.6% of them were born in developing countries. The number of LBW babies is concentrated in two regions of the developing world, Asia (72%) and Africa (22%), and India alone accounts for 40% of LBW births in the developing world [1]. There are nearly eight million LBW infants born in India, which accounts for about 28% of all live births in India [2]. According to the latest WHO and the United Nations International Children’s Emergency Fund (UNICEF) estimates, there is partial data available from approximately 54 countries that include India [2]. The infant mortality rate in India is 37%, and in Telangana state, it stands at 34% [3]. The principal cause of infant mortality in India is LBW, which measures 57% of all causes [4].

Among the several factors that result in LBW, major factors include preterm birth, intrauterine growth restriction (IUGR), or a combination of both pathological and physiological conditions. Most LBW newborns in Western countries are preterm. However, in India, about two-thirds of LBW babies are born at term [5]. Infants with intrauterine growth restriction (IUGR) have greater morbidity and mortality than do appropriately grown, gestation-matched infants. Malnutrition during infancy is a major determinant of LBW because over 40% of LBW babies are malnourished at one year of age. It was also observed that LBW babies have 2.3 times increased risk of mortality due to infections compared to normal birth weight babies [6]. LBW is also an important determinant of newborn and childhood morbidity, especially accountable for neurodevelopmental disorders such as mental retardation and learning disabilities, among others.

Recent evidence indicates that obesity, type 2 diabetes mellitus, and cardiovascular disease are more common among adults who were having IUGR at birth. Studies suggest that these may represent an example of “programming,” in which an insult, when applied at a critical or sensitive stage in development, may result in a lifelong effect on the structure or function of the organism [7]. There are numerous aspects contributing to LBW that include both maternal and fetal factors. Weight at birth is directly influenced by the general level of the health status of the mother. Moreover, the maternal environment is the most important determinant of newborn birth weight.

The factors that are considered potential determinants of LBW include low-income level, resource-limited developing countries, maternal factors such as socioeconomic status, inadequate nutrition, bad obstetric history, less frequent antenatal visits, low pre-pregnancy weight, short maternal stature, hypertension, and endemic infections such as malaria, among others [8]. Maternal risk factors are socially and biologically interrelated. Most are, however, modifiable. The incidence of LBW babies can be lessened if the maternal factors are detected early and managed by simple techniques. Thus, it is necessary to identify factors prevailing in a particular area responsible for LBW. In the present study, we aimed to assess the prevalence of LBW among newborn babies and identify the potential maternal risk factors for LBW in pregnant women attending a tertiary care hospital.

## Materials and methods

This hospital-based cross-sectional study was carried out at Government Medical College and Hospital, Nizamabad, Telangana, India, between June 2016 and May 2017. The study included mothers of 900 newborn babies who gave informed consent to participate, and the study was approved by the institutional ethics committee (IEC/Annexure A-22-06-2016).

Inclusion and exclusion criteria

The inclusion criteria determined were all consecutive live newborn babies born at term and singleton pregnancy, i.e., birth of a single newborn during the delivery. The exclusion criteria included pre-term babies, post-term babies, intrauterine deaths, stillbirths, twins or more pregnancies, and newborns with congenital abnormalities.

A predefined and validated questionnaire was used to collect the data. The questionnaire included demographic and clinical characteristics such as age, religion, address, last menstrual period (LMP), expected delivery date (EDD), parity, birth spacing, pre-pregnancy weight, weight gain during pregnancy (weight gain was calculated by subtracting the weight of the mother at 12 weeks or before from weight of the mother at term considering negligible weight gain up to 12 weeks of gestation), height, mother’s education, mother’s occupation, family income, socioeconomic status (calculated using modified Kuppuswamy’s socioeconomic status scale, revised for 2016), antenatal visits and checkups, obstetric history of the mother especially information about previous stillbirths and abortions and past history of any LBW baby, history of any protein and energy supplementation being taken under the Integrated Child Development Services (ICDS) scheme, history of any strenuous physical activity, personal habits such as smoking (both active and passive and tobacco chewing), alcohol/toddy consumption, maternal infections (fever, malaria, urinary tract infection (UTI), tuberculosis (TB), and bacterial vaginosis), systemic diseases (hypertension and diabetes mellitus), hemoglobin (HB) concentration (estimated within 15 days prior to delivery), and regular consumption/supplementation of iron and folic acid tablets.

Antenatal care was considered adequate if the pregnant woman had a minimum of four antenatal checkups. According to HB concentrations, mild anemia was considered if HB was 10-10.9 g/dL, moderate anemia when HB was 7-9.9 mg/dL, and severe anemia if HB was less than 7 mg/dL. Birth weight was measured within one hour of birth with an electronic weighing machine. Preterm babies were excluded by assessing gestational age with the help of an ultrasound scan, and the gestational maturity of the baby was determined using the new Ballard scoring system.

Statistical analysis

Data were entered and analyzed using Microsoft Office 2007 Excel sheet (Microsoft Corp., Redmond, WA, USA). The total number of births occurring during the study period was recorded, and the prevalence of LBW babies was calculated. Data were presented in descriptive tables, and inferential statistics were done. Chi-square tests were performed to evaluate the association of various maternal risk factors with LBW. A p-value of less than 0.05 was considered statistically significant. Data analysis was carried out using the Statistical Package for the Social Sciences (SPSS) version 16.0 (IBM SPSS Statistics, Armonk, NY, USA) and EPI Info 3.5.1.

## Results

Of the 900 newborn babies included in the study, 327 were identified as LBW babies with a prevalence rate of 36.33%. The occurrence of LBW babies was predominant among mothers who were aged <19 years (62.26%) and >35 years (57.14%). The prevalence of LBW based on maternal age is depicted in Table 1.

**Table 1:** Distribution of LBW babies according to maternal age Chi-square=20.87, df=2, p=0.0001 LBW: low birth weight

Maternal age (years)	Birth weight < 2.5 kg (number (%))	Birth weight > 2.5 kg (number (%))	Total (number (%))
<19	99 (62.26)	60 (37.73)	159 (17.66)
19-35	216 (30)	504 (70)	720 (80)
>35	12 (57.14)	9 (42.85)	21 (2.33)
Total	327 (36.33)	573 (63.66)	900 (100)

Religion-wise, Muslim mothers revealed the highest prevalence (48.57%) of LBW, followed by Hindus (37.71%) and Christians (20%). Others who either were hesitant to reveal their religion or did not belong to the listed religion gave birth to normal babies (100%). The religion-wise distribution of LBW babies is shown in Table 2.

**Table 2:** Religion-wise distribution of LBW babies Chi-square=18.967, df=3, p=0.0001 LBW: low birth weight

Religion	Birth weight < 2.5 kg (number (%))	Birth weight > 2.5 kg (number (%))	Total (number (%))
Hindu	129 (37.71)	213 (62.28)	342 (38)
Muslim	153 (48.57)	162 (51.42)	315 (35)
Christian	45 (20)	180 (80)	225 (25)
Others	0 (0)	18 (100)	18 (2)
Total	327 (36.33)	573 (63.66)	900 (100)

Grand multipara women showed the highest rates (53.70%) of LBW babies. The number of LBW babies in primiparous women was 102 (33.66%), and in multiparous women, the number of LBW babies was 51 (18.68%). The details of parity among the study participants are detailed in Table 3.

**Table 3:** Parity in relation to LBW among the study participants Chi-square=26.55, df=1, p=0.0001 LBW: low birth weight

Parity	Birth weight < 2.5 kg (number (%))	Birth weight > 2.5 kg (number (%))	Total (number (%))
Primipara	102 (33.66)	201 (66.33)	303 (33.66)
Multipara (2-3)	51 (18.68)	222 (81.31)	273 (30.33)
Grand multipara (4 and above)	174 (53.70)	150 (46.29)	324 (36)
Total	327 (36.33)	573 (63.66)	900 (100)

Additionally, LBW was predominantly noticed among newborns (46.66%) with a birth spacing of <18 months. In babies with a birth spacing between 18 and 24 months, 90 (30.61%) revealed LBW, and in whom birth spacing was greater than 24 months, the incidence of LBW was 48 (23.88%). The details of the newborn birth spacing are shown in Table 4.

**Table 4:** Birth spacing in relation to the birth weight of the newborn babies Chi-square=12.11, df=2, p=0.002

Birth spacing (months)	Birth weight < 2.5 kg (number (%))	Birth weight > 2.5 kg (number (%))	Total (number (%))
<18	189 (46.66)	216 (53.33)	405 (45)
18-24	90 (30.61)	204 (69.38)	294 (32.66)
>24	48 (23.88)	153 (76.11)	201 (22.33)
Total	327 (36.33)	573 (63.66)	900 (100)

The number of LBW babies for mothers whose pre-pregnancy weight was less than 40 kg was 237 (94.0%), and for those whose pre-pregnancy weight was more than 40 kg, the number of LBW babies was 90 (13.88%). The weight-wise distribution of newborns based on the mother’s pre-pregnancy weight is depicted in Table 5.

**Table 5:** Weight-wise distribution of newborns in comparison to the mother’s pre-pregnancy weight Chi-square=167.99, df=1, p=0.0001

Pre-pregnancy weight (kg)	Birth weight < 2.5 kg (number (%))	Birth weight > 2.5 kg (number (%))	Total (number (%))
<40	237 (94.04)	15 (5.95)	252 (28)
>40	90 (13.88)	558 (86.11)	648 (72)
Total	327 (36.33)	573 (63.66)	900 (100)

The relationship between the mother’s weight gain during pregnancy and LBW revealed that when the mother’s weight gain during pregnancy was less than 7 kg, there is an increased probability of LBW babies (82.2%) when compared to the weight gain of more than 7 kg during pregnancy (6.59%). The mother’s weight gain during pregnancy and its relation to LBW babies is shown in Table 6.

**Table 6:** Relationship between mother’s pre-pregnancy weight gain and LBW babies Chi-square=176.91, df=1, p=0.0001 LBW: low birth weight

Weight gained during pregnancy (kg)	Birth weight < 2.5 kg (number (%))	Birth weight > 2.5 kg (number (%))	Total (number (%))
<7	291 (82.20)	63 (17.79)	354 (39.33)
>7	36 (6.59)	510 (93.40)	546 (60.66)
Total	327 (36.33)	573 (63.66)	900 (100)

The number of LBW babies born to mothers whose height was less than 145 cm was 318 (83.46%), and the number of LBW babies born to mothers whose height was more than 145 cm was nine (1.73%). The incidence of LBW newborns in relation to the mother’s height is depicted in Table 7.

**Table 7:** Incidence of LBW among newborns in relation to the mother’s height Chi-square=211.48, df=1, p=0.0001 LBW: low birth weight

Height of the mother (cm)	Birth weight < 2.5 kg (number (%))	Birth weight > 2.5 kg (number (%))	Total (number (%))
<145	318 (83.46)	63 (16.533)	381 (42.33)
>145	9 (1.73)	510 (98.26)	519 (57.66)
Total	327 (36.33)	573 (63.66)	900 (100)

A majority of LBW babies were born to mothers who were illiterate (105, 43.75%), and when the mother’s education was up to primary school, the incidence of LBW was 198 (41.25%). However, when the mother’s education was up to high school (21, 17.50%) and pre-university (3, 5%), LBW incidences were noted to be least as shown in Table 8.

**Table 8:** Relationship between the mother’s educational level and the incidence of LBW Chi-square=18.196, df=3, p=0.0001 LBW: low birth weight

Mother’s education	Birth weight < 2.5 kg (number (%))	Birth weight > 2.5 kg (number (%))	Total (number (%))
Illiterate	105 (43.75)	135 (56.25)	240 (26.66)
Primary school	198 (41.25)	282 (58.75)	480 (53.33)
High school	21 (17.50)	99 (82.50)	120 (13.33)
Pre-university	3 (5)	57 (95)	60 (6.66)
Total	327 (36.33)	573 (63.66)	900 (100)

When mothers were agricultural workers, the number of LBW babies born was highest at 132 (63.76%). Among the daily wage labor working mothers, the number of LBW babies born was 150 (51.02%). When mothers were under service type of occupation, the number of LBW babies born was 24 (11.11%), and when mothers were homemakers, the number of LBW babies born was 21 (11.47%) as shown in Table 9.

**Table 9:** Mother’s occupation and its relationship with LBW Chi-square=67.685, df=3, p=0.0001 LBW: low birth weight

Occupation of the mother	Birth weight < 2.5 kg (number (%))	Birth weight > 2.5 kg (number (%))	Total (number (%))
Agricultural worker	132 (63.76)	75 (36.23)	207 (23)
Daily wage laborer	150 (51.02)	144 (48.97)	294 (32.66)
Service	24 (11.11)	192 (88.88)	216 (24)
Homemaker	21 (11.47)	162 (88.52)	183 (20.33)
Total	327 (36.33)	573 (63.66)	900 (100)

Mothers belonging to the lower socioeconomic class revealed the highest number of LBW babies (246, 52.90%) when compared to those belonging to the middle class (72, 28.57%) and the upper middle class (9, 4.91%) as depicted in Table 10.

**Table 10:** Incidence of LBW in comparison with the socioeconomic status Chi-square=46.610, df=2, p=0.0001 LBW: low birth weight

Socioeconomic class	Birth weight < 2.5 kg (number (%))	Birth weight > 2.5 kg (number (%))	Total (number (%))
Upper middle	9 (4.91)	174 (95.08)	183 (20.33)
Middle	72 (28.57)	180 (71.42)	252 (28)
Lower	246 (52.90)	146 (31.39)	465 (51.66)
Total	327 (36.33)	573 (63.66)	900 (100)

Other maternal factors that could predispose to LBW included the number of LBW babies in mothers with antenatal visits of less than four times (315, 59.65%), and in mothers with antenatal visits of more than four times, it stood at 12 (3.22%) as shown in Table 11.

**Table 11:** Number of antenatal visits in comparison with LBW Chi-square=100.15, df=1, p=0.0001 LBW: low birth weight

Antenatal visits (number)	Birth weight < 2.5 kg (number (%))	Birth weight > 2.5 kg (number (%))	Total (number (%))
<4	315 (59.65)	213 (40.34)	528 (58.66)
>4	12 (3.22)	360 (96.77)	372 (41.33)
Total	327 (36.33)	573 (63.66)	900 (100)

In mothers with blood HB concentrations less than 7 mg/dL (severe anemia), 162 (100%) delivered LBW babies. Ninety-nine (22.60%) newborns have LBW and were delivered by mothers with blood HB concentrations between 7 and 9.9 mg/dL (moderate anemia). Interestingly, mothers with blood HB concentrations greater than 9.9 mg/dL delivered 66 (22%) LBW newborns as shown in Table 12.

**Table 12:** Mother’s blood HB concentrations in relation to LBW Chi-square=115.40, df=2, p=0.0001 HB: hemoglobin, LBW: low birth weight

Mother’s blood HB concentrations (mg/dL)	Birth weight < 2.5 kg (number (%))	Birth weight > 2.5 kg (number (%))	Total (number (%))
<7	162 (100)	0 (0)	162 (18)
7-9.9	99 (22.60)	339 (77.39)	438 (48.66)
>9.9	66 (22)	234 (78)	300 (33.33)
Total	327 (36.33)	573 (63.66)	900 (100)

When the history of strenuous physical activities among mothers was assessed, it was noted that strenuous physical activity during pregnancy revealed susceptibility to delivering LBW newborns in 285 (48.46%). However, the incidence of LBW babies was considerably less (13.46%) when the mother had no history of strenuous physical activity during the pregnancy period as shown in Table 13.

**Table 13:** Strenuous physical activity during pregnancy and its relation to LBW Chi-square=35.99, df=1, p=0.0001 LBW: low birth weight

History of strenuous physical activity during pregnancy	Birth weight < 2.5 kg (number (%))	Birth weight > 2.5 kg (number (%))	Total (number (%))
Yes	285 (48.46)	303 (51.53)	588 (65.33)
No	42 (13.46)	270 (86.53)	312 (34.66)
Total	327 (36.33)	573 (63.66)	900 (100)

The number of LBW babies born among mothers who had a history of tobacco smoking and chewing tobacco during pregnancy was higher at 192 (91.42%) compared to those who did not have such habits (135, 19.56%) as shown in Table 14.

**Table 14:** Tobacco smoking and chewing habit in relation to LBW Chi-square=119.81, df=1, p=0.0001 LBW: low birth weight

Smoking/chewing tobacco	Birth weight < 2.5 kg (number (%))	Birth weight > 2.5 kg (number (%))	Total (number (%))
Yes	192 (91.42)	18 (8.57)	210 (23.33)
No	135 (19.56)	555 (80.43)	690 (76.66)
Total	327 (36.33)	573 (63.66)	900 (100)

The number of LBW babies in mothers who had a history of consumption of alcohol and locally brewed toddy during pregnancy was 192 (66.66%) compared to mothers who did not have such habits (135, 22.05%) as shown in Table 15.

**Table 15:** Alcohol consumption in relation to LBW Chi-square=56.155, df=1, p=0.0001 LBW: low birth weight

Alcohol/toddy consumption	Birth weight < 2.5 kg (number (%))	Birth weight > 2.5 kg (number (%))	Total (number (%))
Yes	192 (66.66)	96 (33.33)	288 (32)
No	135 (22.05)	477 (77.94)	612 (68)
Total	327 (36.33)	573 (63.66)	900 (100)

The number of LBW babies born in mothers who had taken protein and calorie supplementation provided under the government scheme was 183 (36.52%). Interestingly, women who did not receive protein and calorie supplementation also revealed a similar incidence of LBW newborns (144, 36.09%) as shown in Table 16.

**Table 16:** Protein and calorie supplementation in relation to LBW Chi-square=0.06, df=1, p=0.938 LBW: low birth weight

Protein and calories supplementation	Birth weight < 2.5 kg (number (%))	Birth weight > 2.5 kg (number (%))	Total (number (%))
Yes	183 (36.52)	318 (63.47)	501 (55.66)
No	144 (36.09)	255 (63.90)	399 (44.33)
Total	327 (36.33)	573 (63.66)	900 (100)

Conversely, newborns of mothers who were supplemented with iron and folic acid had low rates of LBW babies compared to mothers who did not take any such supplementation as shown in Table 17.

**Table 17:** Iron and folic acid supplementation in relation to LBW Chi-square=48.7, df=1, p=0.0001 LBW: low birth weight

Iron and folic acid supplementation	Birth weight < 2.5 kg (number (%))	Birth weight > 2.5 kg (number (%))	Total (number (%))
No	186 (64.58)	102 (35.41)	288 (32)
Yes	141 (23.03)	471 (76.96)	612 (68)
Total	327 (36.33)	573 (63.66)	900 (100)

In mothers with a past history of abortions, the number of LBW babies was 30 (35.71%). A past history of stillbirths revealed the highest incidence of LBW babies (51, 51.51%). Additionally, a past history of neonatal deaths (33.33%) and a previous history of birth of LBW babies (30.43%) had no significant relation to LBW babies. The details of the mother’s past obstetric history are detailed in Table 18.

**Table 18:** Obstetric history and its relationship with LBW Chi-square=3.379, df=3, p=0.337 LBW: low birth weight

Bad obstetric history	Birth weight < 2.5 kg (number (%))	Birth weight > 2.5 kg (number (%))	Total (number (%))
Abortions	30 (35.71)	54 (64.28)	84 (25.22)
Stillbirths	51 (51.51)	48 (48.48)	99 (29.72)
Neonatal deaths	27 (33.33)	54 (66.66)	81 (24.32)
Previous LBW baby	21 (30.43)	48 (69.56)	69 (20.72)
Total	129 (38.73)	204 (61.26)	333 (100)

The incidence of LBW babies in mothers who had a fever during pregnancy was 21 (24.1%), and those who had malaria was 27 (31.03%), urinary tract infection was 87 (34.93%), and bacterial vaginosis was 39 (35.13%). A high percentage of LBW babies was noticed among mothers who had tuberculosis (75%) as shown in Table 19.

**Table 19:** Maternal infections during pregnancy and their relationship with LBW Chi-square=4.43, df=4, p=0.351 LBW: low birth weight

Maternal infections	Birth weight < 2.5 kg (number (%))	Birth weight > 2.5 kg (number (%))	Total (number (%))
Fever	21 (24.13)	66 (75.86)	87 (15.93)
Malaria	27 (31.03)	60 (68.96)	87 (15.93)
Tuberculosis	9 (75)	3 (25)	12 (2.19)
Urinary tract infection	87 (34.93)	162 (65.06)	249 (45.60)
Bacterial vaginosis	39 (35.13)	72 (64.86)	111 (20.32)
Total	183 (33.51)	363 (66.48)	546 (100)

The highest percentage of LBW babies were born in mothers with chronic hypertension, preeclampsia, and eclampsia (47.6%), followed by those with diabetes and gestational diabetes (38.46%), bronchial asthma (33.33%), sickle cell anemia (32%), and heart diseases (30.76%) as shown in Table 20.

**Table 20:** Relationship between LBW babies and mother’s systemic illnesses Chi-square=1.854, df=4, p=0.763 LBW: low birth weight

Systemic diseases	Birth weight < 2.5 kg (number (%))	Birth weight > 2.5 kg (number (%))	Total (number (%))
Chronic hypertension, preeclampsia, eclampsia	30 (47.61)	33 (52.38)	63 (17.21)
Heart disease	24 (30.76)	54 (69.23)	78 (21.31)
Diabetes, gestational diabetes mellitus	30 (38.46)	48 (61.53)	78 (21.31)
Bronchial asthma	24 (33.33)	48 (66.66)	72 (19.67)
Sickle cell anemia	24 (32)	51 (68)	75 (20.49)
Total	132 (36.06)	234 (63.93)	366 (100)

The mother’s age (p=0.002), height (p=0.0001), pre-pregnancy weight (p=0.0001), weight gain during pregnancy (p=0.0001), smoking and alcohol consumption (p=0.0001), education (p=0.0001), occupation (p=0.0001), socioeconomic status (p=0.0001), history of strenuous physical activity (p=0.0001), blood HB concentrations (p=0.0001), number of antenatal visits (p=0.0001), and parity (p=0.0001) are significantly related to LBW. Interestingly, the presence of systemic illness (p=0.763), protein and calorie supplementation (p=0.938), infections during pregnancy (p=0.351), and bad obstetrics history (p=0.337) did not correlate with LBW.

## Discussion

Birth weight is the single most important marker for perinatal and neonatal outcomes [9]. LBW is a significant determinant of neurodevelopmental disabilities and adult-onset diseases such as type 2 diabetes mellitus and ischemic heart disease [7]. The etiology of LBW is multifactorial, and therefore, this study was undertaken to know the influence of various maternal and social factors on the birth weight of newborns.

In the present study, the prevalence of LBW was high at 36.3% compared to district-level household and facility survey data, according to which the percentage of LBW was 7.1% in the study area. Moreover, the state-wise data of LBW stood at 8.6% in Telangana state as estimated by the local government [10]. According to National Family Health Survey (NFHS-5) data, LBW in India was recorded at 18% [11]. Despite a decreasing trend noted in the earlier surveys (NFHS-1: 25.2%, NFHS-2: 22.9%, NFHS-3: 20.9%), an increase of 2% was evident in comparison to the NFHS-4 survey (16%) [12]. The prevalence in the present study is comparable to previous studies from India conducted by Noor et al. [13] and Juneja et al. [14], who reported prevalence rates of 36.8% and 40%, respectively. Interestingly, the prevalence rates were extremely high when compared to the national prevalence (18%) as provided by the Indian government [11]. The high prevalence rate noted in this study can be because Government Medical College, Nizamabad, is a referral hospital that receives high-risk pregnancy cases from local area hospitals, and mothers who undergo delivery mostly belong to the low socioeconomic class.

The present study showed a higher percentage of LBW babies among mothers who were younger than 19 years of age and those who were aged over 35 years. The study results were consistent with the findings of Agarwal et al. [15] and Kaur et al. [16] but were contradictory to the results obtained by Prudhivi et al. [17], who did not find any significant association between maternal age and LBW. Early age of marriage is an established custom, especially in rural and illiterate families in India and other developing and poor nations. Teenage girls are physically and physiologically naive for reproduction, and this results in LBW babies. Women aged above 35 years were mostly grand multiparas with increased incidences of complications such as hypertension and diabetes, which potentially predisposed them to deliver LBW babies.

In the present study, religion was found to be significantly associated with LBW babies with a higher percentage of LBW babies noted in Muslim mothers. However, the results of the study by Raghunath et al. [18] revealed a higher rate of LBW babies among Hindu mothers. Conversely, studies by Kaur et al. [16] and Prudhivi et al. [17] found that religion did not have a significant effect on LBW babies. The higher percentage of LBW babies in Muslim mothers in this study may be due to more percentage of the Muslim population belonging to low socioeconomic status.

A significant association was found between grand multipara and primipara status with the incidence of LBW. This is consistent with the findings of Kaur et al. [16] and Prudhivi et al. [17], who noted a significant association of LBW with parity. On the contrary, the present study results were contradictory to the findings of Sumana et al. [19] who found no association of LBW with parity.

The reason for primiparas being increasingly susceptible to delivering LBW babies can be compromised uteroplacental blood flow, which is responsible for delivering oxygen and nutrients to the fetus. Additionally, there may be structural factors that limit uterine capacity in the first pregnancy, after which the uterine size increases. Furthermore, firstborn infants may be exposed to a different maternal immune environment, contributing to relative growth restriction, compared to subsequent pregnancies with consistent paternity. Grand multipara had a higher probability of pregnancy-related complications, and advancing maternal age among them would have also been an additional confounding factor leading to LBW.

Birth spacing of fewer than 18 months is significantly associated with LBW. This is consistent with the findings of Kaur et al. [16] and Sumana et al. [19]. However, the results of the study by Raghunath et al. [18] did not find a relationship between LBW and birth spacing. The higher percentage of LBW in mothers with shorter birth spacing may be explained by the deterioration of nutritional status during pregnancy.

Pre-pregnancy weight of less than 40 kg was significantly associated with LBW in the present study. This was consistent with the study findings of Prudhivi et al. [17]. However, the results contradicted the study findings of Raghunath et al. [18], which did not show any such relationship. A higher percentage of LBW in mothers with less pre-pregnancy weight can be due to malnourishment and fewer body stocks of proteins and fats.

Weight gained during pregnancy of fewer than 7 kg was significantly associated with LBW. This is consistent with the study findings of Rajashree et al. [20] and Raghunath et al. [18]. Less weight gain during pregnancy can be due to factors such as inadequate intake of nutritious food and hard manual labor work during pregnancy.

Mother’s height of fewer than 145 cm significantly correlated with LBW. This is consistent with the study findings of Sumana et al. [19], Prudhivi et al. [17], and Agarwal et al. [15]. However, the findings of Soujanya et al. [21] found no such relationship. Maternal height is influenced by heritable factors, environmental factors, and others. Undernutrition appears to play an influential role in the development of LBW babies.

In the present study, a higher percentage of LBW babies were born to illiterate mothers, followed by those who were educated up to primary school and high school. Similar findings were noted in studies by Kaur et al. [16], Raghunath et al. [18], Sumana et al. [19], and Prudhivi et al. [17]. However, Reddy et al. [22], in their study, found no such relationship. The low percentage of LBW in educated mothers may be because of increased awareness about healthcare services and better pregnancy care.

LBW was predominantly found among mothers who were agricultural workers. Similar findings were reported by Rajashree et al. [20] and Shahnawaz et al. [23]. However, no such association was observed in the study by Reddy et al. [22]. The increased prevalence of LBW in agricultural workers may have been due to increased physical activity and less rest.

Low socioeconomic status was noted to predispose women to deliver LBW babies. Identical results were noted in the studies by Agarwal et al. [15], Raghunath et al. [18], and Bendhari et al. [24]. On the contrary, Shahnawaz et al. [23], in their study, found no relationship between the mother’s socioeconomic status and LBW. Interestingly, socioeconomic status does not have an independent effect on birth weight. However, it appears that low socioeconomic status can potentially predispose pregnant women to infections, nutritional deficiency, and others that may result in LBW in newborns.

The number of antenatal visits was also found to influence birth weight as evidenced by the high prevalence of LBW among mothers who reported less than four antenatal visits during pregnancy. Similar findings were noted in a previous study by Kaur et al. [16]. Fewer LBW rates when proper antenatal care was given can be due to treatment of modifiable risk factors such as infections, anemia, and hypertension.

Severe anemia appears to be a potential risk factor for LBW, and similar conclusions were drawn in the studies by Gupta et al. [25], Raghunath et al. [18], and Kaur et al. [16]. Given that maternal anemia limits oxygen uptake and thereby decreases oxygen delivery, it results in fetal growth restriction.

The percentage of LBW babies (36.5%) born to mothers who had taken protein and calorie supplementation under the government scheme was similar (36.5%) to those who had not received such supplementation. However, a previous study from Sudan revealed that maternal undernutrition could be a potential risk factor for LBW [26].

In the present study, a higher percentage of LBW infants were born when the mother had a history of strenuous physical activity. These results were similar to those reported by Kaur et al. [16]. Strenuous physical activity during pregnancy can alter the physiological balance in mothers with a marginal nutritional deficiency, which can lead to LBW babies.

Tobacco smoking and chewing habits during pregnancy were noted to predispose mothers to deliver LBW babies. Similar findings were reported by Agarwal et al. [15], Bendhari et al. [24], and Johnson et al. [27]. On the contrary, the findings of Louis et al. [28] and Raghunath et al. [18] did not find any such association. Nicotine can significantly affect placental vasculature causing hypoxia and fetal growth retardation.

Alcohol/toddy consumption during pregnancy was found to predispose mothers to deliver LBW babies. A similar association was noted in a study by Nykjaer et al. [29]. On the contrary, a study by Louis et al. [28] found no such effect. Superstitious beliefs/misconceptions that drinking toddy during pregnancy can lead to the birth of a fair baby and increase amniotic fluid could be the reason for such a habit among women of this region, which could invariably predispose them to LBW babies.

In the present study, a high percentage of LBW babies were born when the mother had not taken iron and folic acid supplementation during pregnancy. Similar findings were reported in a study from Nepal, which found that iron and folic supplementation and the number of antenatal visits were associated with LBW [30].

A bad obstetric history was found not to be a significant contributory factor for LBW babies. This is consistent with the study findings of Negi et al. [31] and Bendhari et al. [24]. However, a study reported by Sumana et al. [19] found a significant association between bad obstetric history and LBW.

The current study results found no significant association between maternal infections and LBW as noticed in a study by Soujanya et al. [21]. Conversely, the study findings of Saini et al. [32] revealed a significant association between maternal infections and LBW. In an interesting observation, mothers who had tuberculosis during pregnancy had a high percentage of LBW babies (75%) compared to the others. Similar findings were noticed in a study by Bates et al. [33] and Fekadu et al. [34].

A high percentage of LBW babies was noted among mothers who had chronic hypertension, preeclampsia, and eclampsia (47.60%) compared to those with other systemic diseases such as diabetes, bronchial asthma, sickle cell anemia, and heart diseases during pregnancy [35].

An assessment of the results of this study and other studies points to the fact that there is scarce data on the prevalence of LBW and associated modifiable and unmodifiable maternal factors, especially in poor and developing nations such as Ghana and India, respectively [36].

Study limitations

The present study was carried out at a government hospital, where the facilities are offered free of cost and may not be utilized by all sections of the community. Therefore, study results will not necessarily reflect the population scenario. Another factor that could have caused bias in the study results is imprecise information regarding a few factors attributed to the fact that the majority of mothers were illiterate and belonged to a rural area and did not have appropriate medical records.

## Conclusions

The results of this study provide empirical support for the potential links that exist between maternal health and infant birth weight. A significant association was found between LBW and maternal factors such as age, weight, height, religion, literacy levels, occupation of mothers, family income, socioeconomic status, antenatal visits/care, strenuous physical activity during pregnancy, smoking/tobacco chewing, alcohol/toddy consumption, and iron and folic acid supplementation, among others. Increased public health awareness programs along with integrated and complementary strategies are required to address the issue of LBW newborns. Interventional programs should be encouraged not only in health sectors but also in all those areas concerned with social development and social welfare. Adolescents should be educated regarding marriage age and nutritious food intake during pregnancy, and the deleterious effects of early teenage marriages. There appear to be multiple maternal factors that affect the newborn’s birth weight. This suggests that a single factor cannot be attributed to the occurrence of LBW babies. Health education, socioeconomic development, maternal nutrition, and increasing availability and accessibility of health services during pregnancy are equally important to minimize the incidences of LBW among newborns.
